# Hospital and Institutional News

**Published:** 1915-07-17

**Authors:** 


### HOSPITAL AND INSTITUTIONAL NEWS.

### THE CONTROL OF NURSING HOMES.

The London County Council's (General Powers) Bill has now passed its third reading in the House of Commons. The Bill was originally introduced last year and contained a variety of proposals, the most notable of them being the registration and control of lying-in homes and of establishments for nursing and special treatment. For a long time the Bill made no progress, owing to opposition which was offered to one section dealing with the control of premises upon which celluloid or cinematograph films were stored or manufactured. Negotiations proceeded for a considerable time, but a settlement was not arrived at, and in order to secure progress with the other important and unopposed parts of the Bill the suggestion was made that the Bill should be divided and the celluloid and cinematograph films proposal should form the subject of a separate measure. This course has been approved by the House of Commons, and the general Bill dealing with a variety of subjects, including nursing establishments and lying-in homes, was passed by the House of Commons at the end of last week.

### ST. BARTHOLOMEW'S HOSPITAL STAFF AND WAR SERVICE.

At one time there existed considerable doubt at St. Bartholomew's Hospital as to its position if the students qualifying were commandeered by the R.A.M.C. There might have been in that case, it was feared, some difficulty in filling the appointments on the staff, but a compromise has been arrived at which should remove the likelihood of a dearth of doctors occurring at the hospital. The Director-General of Medical Services was approached, and he has formulated a scheme whereby the members of the junior staff may receive temporary honorary commissions. On nomination to the junior staff the men wishing to hold commissions shall apply to the Dean of the Medical School, who will give them a certificate of qualification to take to the War Office. The men will be given honorary commissions and an allowance for their outfit. They will then be seconded for a period of three months in order to act as residents at the hospital. During this three months they are liable to be called up for active service at forty-eight hours' notice. Before gazetting every man will sign a contract that he will serve for one year at home or abroad from the time he finishes his resident appointment. The advantages of the scheme are that the civil hospital is provided with an adequate staff of medical officers, and the War Office is supplied with a better-trained man than would be the case in accepting him immediately after his qualification.

### WAR LOAN GRATUITIES.

The members of the committee of the Gravesend Hospital have presented each employee, who has been in the service of the hospital at least three months, with a small holding in the War Loan as a slight recognition of extra services rendered during a particularly busy time. It is interesting to add that, while the object in view of the committee was the twofold one of encouraging the success of the War Loan and at the same time of giving a mark of their appreciation for the extra and ungrudging service given by their employees since the war began, the money providing for the "gratuity" has not come out of the general funds of the hospital. Put briefly, there has been in existence and in the hands of members of the committee what may be described as a reserve fund for the general purposes of the hospital. The rewarding of loyal and additional service was felt to be within the intentions of this fund, and some forty employees of the hospital have benefited by it. While a more substantial gift has been made to certain of the higher officers, each of the rank and file has received a scrip, bearing interest from June 30 last, which they are free to ask the hospital's secretary, Mr. Pearson, to cash at any time. It is hoped that the present favourable reception of the scheme on the part of the hospital's employees may lead to further "investments" every three months. The plan, at all events, deserves to be brought to the notice of all hospital managers anxious, as some of them are, to know what other hospitals are doing in relation to the policy of giving a war bonus to this or that section of the staff. It is apparently correct to state that while the nurses of one or two institutions have had their salaries raised since the war, "war bonuses" seem to have been limited generally to the junior members of the administration.

### "LATEST INSTALLATIONS IN HOSPITAL EQUIPMENT."

An important new section, as our readers will have noticed, started in The Hospital last week in the form of a column entitled "Latest Installations in Hospital Equipment: What Others are Doing." This is the beginning of what, with the continued co-operation of our readers, will form an invaluable file for all administrative officers considering the installation of every new kind of equipment from, say, a patent self-balancing suspended hydro-extractor for the laundry, to a ward window, and anxious to know what other institutions have lately done when in the like case. A very large part of the correspondence of an up-to-date superintendent in a well-organised hospital is devoted to answering questions of this kind from other secretaries all over the country. *No file of the experience gained has so far been available.* In response to a widely-expressed demand, our column, devoted to recording the adoption of new installations, will supply one. It will appear in every issue, and always be available for reference. In this way, without asking questions of one another, each hospital secretary and executive officer can see at a glance which institution has adopted what equipment. The statements in the column, in order to be as terse and accessible as possible, confine themselves to statements of fact—the name of the institution which has installed this or that plant; the nature of the plant; and the name and address of the firm which has supplied it. With this information provided for him, each institutional officer will have all the threads in his hands for pursuing inquiries in any practical direction he chooses. We shall always be pleased, on request, to supply information concerning installations and firms mentioned on previous occasions in this column; so that if any officer wants to know what institutions have lately been adopting what kind of water-softening device, ward lockers, etc., a card to us will bring him all references to the matter which the installations column has contained. To keep the file complete and up to date, we invite the co-operation of all secretaries and executive officers in supplying us with information concerning any new form of equipment they have adopted. In this way all will benefit through the file which all jointly combine to maintain for ready reference in The Hospital. Much time and correspondence will be saved to superintendents by the simple plan now successfully launched.

### THE SALARIES OF HEALTH VISITORS.

At the end of last year the London County Council considered whether a portion of the salaries of health visitors, employed in maternity and infant welfare schemes, which is not borne by the funds of local sanitary authorities in London, should be met out of the Local Government Board's grant. There was then, and there is now, no immediate likelihood of any surplus being available to meet such payments by the County Council, and the Council expressed the opinion that contributions towards the salaries of health visitors should be borne by national funds and not by the Council. The Local Government Board has now intimated that it will be prepared to pay out of the grant for maternity and child welfare work one half of the salaries of infant and maternity health visitors throughout the Metropolis.

### MEDICAL ATTENDANCE FOR THE SOLDIER ON FURLOUGH.

Readers of The Hospital will recall how, since the early days of the war, we have persistently pressed for the establishment of a system under which proper arrangements would be made for the medical attendance of soldiers on furlough. The difficulties in the way are obvious enough, and as we have already discussed and disposed of them we need not repeat that side of the question. It is gratifying, however, to note an announcement on the subject in Parliament, which was made in the course of the debate on Monday last. Briefly put, this resolved itself into an announcement by Mr. G. H. Roberts that the Insurance Commissioners, in co-operation with the War Office, had secured medical attendance to the soldier on furlough. Our advocacy, then, has been successful. We propose this week to limit ourselves to this satisfactory announcement, and to defer until next week a detailed consideration of practical measures proposed to carry out the scheme. In the course of the debate many interesting points were touched upon which deserve fuller treatment than this week we can find space to give. The content and value of the proposals will, however, be dealt with fully in The Hospital next week.

### MARGARINE AS AN ECONOMY.

The efforts made by Boards of Guardians, referred to in an article in our columns last week, to economise on food supplies has led many of them to propose the substitution of margarine for butter. In most cases the proposal has met with approval, but not without opposition. Conflict of opinion as to the dietetic value of these two foods also exists among medical authorities. Some assert that margarine is equal as a food to butter; others say it is less digestible, that it interferes with the supply of calcium salts to the tissues, and that the vegetable oils contained in it do not play the same part in the metabolism of the body as the animal fats in butter. The practical man, after weighing the evidence on the one side against that on the other, and testing the substances under dispute in his own alimentary system, will probably come to the conclusion that the advocates of margarine are right. He may make the reservation that he prefers the flavour of butter, but even here the difference to most palates, if a good brand of margarine is chosen, is very slight. The saving is very great. At present retail prices it is the difference between 1s. 4d. and 1s. 6d. a lb. and 8d. or 9d., and it is permissible to balance this against a slight loss of flavour. One saying which is heard at most Guardians' discussions on the subject is that good margarine is better than inferior butter. This is perfectly true, and when margarine is first introduced the Guardians usually specify brands with a good reputation and pay a fair price. As time goes on, however, the tendency is gradually to lower the standard and to obtain inferior and cheaper brands. This is indefensible, and forms the grounds for much of the opposition to the introduction of margarine into Poor-Law institutions. Cheap brands of margarine are unpleasant in taste, wasteful in use, and difficult of digestion.

### THE CLOSING OF POOR-LAW INSTITUTIONS.

Since the beginning of the war there has been a marked reduction in the number of the inmates of Poor-Law Institutions. Mr. W. Williams, the Local Government Board Inspector, pointed out to the Llanfyllin Guardians that in South Wales two institutions had already been closed, and the inmates boarded out at a third of the cost required to keep them in the institutions closed. He said that if the reduction which had already occurred at Llanfyllin continued it would be necessary for the Guardians to consider the advisability of closing the institution. There were more workhouses in Montgomeryshire than were needed, but attempts to secure co-operation between the Unions with a view to a reduction in the number of institutions had failed! The failure to secure the assistance of the Guardians must not be allowed to stand in the way of an obvious economy, which will also tend to efficiency of treatment. If necessary, the Local Government must use its compulsory powers to bring about the amalgamation of Unions. Even in the probable event of the end of the war being followed by an increase in the number of poor persons chargeable to the Unions, this amalgamation and concentration of effort will enable the provision for destitution to be carried out more economically and more efficiently.

### THE PUNISHMENT OF REFRACTORY INSURED PATIENTS.

In our issue of June 12 we drew attention to a discussion, at a meeting of the Huddersfield Insurance Committee, on the right of a medical officer of a sanatorium to discharge a patient for refusing to carry out the treatment ordered. Another point closely related to this has recently been discussed at a meeting of the Trade Union Approved Societies' Association in Manchester. The question raised was whether an Approved Society has the right to penalise members who decline to carry out the medical instructions given them, by refusing to pay sick benefit. The Chairman of the meeting drew attention to a case where a patient had been discharged from a sanatorium for refusing to obey the medical officer's orders, and where the Society had continued to pay sick benefit. Another delegate pointed out that similar cases had occurred in connection with other forms of sickness. No decision was arrived at by the meeting, but the matter is obviously one of great importance, and one of which more will be heard. Can there be any doubt that in any case where an insured patient unreasonably refuses to carry out his doctor's instructions, if those instructions are in accordance with generally-accepted medical opinion, the patient should be deprived of sick benefit?

### THE POOLE GUARDIANS AND THEIR MEDICAL OFFICER.

In a letter to the Poole Guardians Dr. Gardner Robinson, the medical officer of the workhouse, draws attention to the very unsatisfactory conditions of his appointment. He submits figures showing that he has to expend £45 per annum on drugs out of a salary of £75, and he states that the amount of actual medical work has more than doubled since his appointment, whilst the clerical work devolving upon him as a result of the requirements of the Poor-Law Institutions Order is enormous. He further points out that this clerical work is of such a character that it cannot be delegated to a nurse, but could be carried out by a clerk acting under his personal supervision. The matter was referred to the Finance Committee to consider an increase in Dr. Robinson's salary. The salary is certainly too low, and should be increased, but this in itself will not suffice. The system of contracting with the medical offices of an institution to supply drugs is a pernicious one, and should be abolished. It was strongly condemned by the Royal Commission on the Poor Laws, and nothing can be said in its favour. As we have said repeatedly in discussing the Poor-Law Institutions Order, the clerical work required by it should not be placed upon the medical officer. Dr. Robinson's suggestion of a clerk acting under the direct supervision of the medical officer is the right solution.

### THE HOSPITAL SATURDAY FUND AND ALIEN INSTRUMENT-MAKERS.

Sir T. Vezey Strong, at a recent meeting of the Board of Delegates of the Hospital Saturday Fund Association, of which he is chairman, read a statement from the Executive Committee with regard to members of the official staff who may be desirous of joining His Majesty's Forces. Nine members of the staff had expressed their willingness to join the Colours, and a junior clerk, Mr. C. Axworthy, has already enlisted. The Board, in recognising the loyalty of the staff, pledged themselves to support any voluntary effort made on behalf of these employees, because to continue payment of their salaries from the Fund would be contrary to the articles of association. The Board adopted the following resolution of the Surgical Appliance Committee: "In view of the inhuman and barbarous methods of German warfare this Committee is of opinion that the Board of Delegates of this Fund will no longer approve of grants being made towards the cost of surgical appliances made by enemy aliens; and that grants toward the cost of appliances made by British subjects of German or Austrian origin should only be made after the status of the makers has been carefully investigated." The opening words of the resolution weaken it. The decision of the delegates is commendable, but the wording is unfortunate, and is open to misunderstanding.

### A REDUCTION IN A DRUG ACCOUNT.

The letter appearing in this issue of The Hospital furnishes another example of an institution which is stated to have reduced its drug bill in war-time. In face of the colossal advances in the prices of many commonly prescribed drugs this is a very remarkable fact. Obviously it is due either to a fundamental change in the character and in the amount of the drugs prescribed, or to a considerable reduction in the quantities prescribed. The three causes may have been operating. As the correspondent intimates, the extent to which an institutional pharmacist can effect economies largely depends upon the medical staff. But the keen pharmacist will keep a watchful eye on the prescriptions, and if he finds that a prescriber habitually orders costly drugs, will suggest, in the most tactful manner possible, that if cheaper drugs are available, which, in the prescriber's opinion, would give equally good results, these should be substituted. Admittedly the task is a difficult one for the dispenser, since he is not a therapeutist; but in the course of his training as a pharmacist he must necessarily have acquired much useful information with regard to parallel drugs which would enable him to be of considerable assistance to a medical man desirous of practising economy. It is not expected of the doctor to follow the course of the markets as systematically as does the dispenser, and until the prescriber is definitely told that he is ordering expensive drugs he is often quite ignorant of the fact.

### A CONFERENCE OF HOSPITAL DISPENSERS?

The professional Committee that was appointed by the Government early in the war to advise medical men with regard to drugs that are scarce in consequence of the cutting off of supplies from Germany and Austria has twice issued lists of drugs that should be prescribed sparingly, and institutional doctors have, no doubt, co-operated with that Committee with the twofold object of effecting economies and husbanding the drug supply of the country. But there is a limit to the extent to which economy can be practised, for it is not only German drugs that have advanced in price; those who read the report of the drug market which appears in The Hospital are aware that the tendency of the prices of nearly all drugs is in an upward direction. Under these circumstances it is really very difficult to understand how a hospital drug account can be reduced in war-time if there was not considerable room for economy in time of peace. In all hospitals pharmacists are, of course, making special efforts just now to keep expenses as low as possible, but few of them aspire to bring them below last year's results. Much good could be done by a conference of hospital dispensers on this very subject. There is, in fact, no reason why it should not be definitely suggested that an early date should be fixed for a meeting of institutional pharmacists for the purpose of considering what means can best be adopted for economising on the drug bills, at any rate during the continuance of the war.

### A NEW DELICACY IN SICK-ROOM COOKERY.

Now that strawberries are cheap it is a good opportunity to remind those responsible for sick-room cookery that a generally unknown delicacy, suitable for invalids who are precluded from enjoying fruit containing pips, is economically obtainable by any housekeeper or cook who is keen enough on her work not to grudge the care and time necessary for correctly carrying out the following recipe: To every 1 lb. of fresh strawberries add ½ lb. of preserving sugar. Before placing in a saucepan, with the addition of a very little water, rub the pan well over with olive oil, (a genuine tip which will effectively prevent the pan from burning). Having placed in the oiled pan the strawberries, preserving sugar, and a little water, in the above proportions, bring the mixture to the boil till it becomes a thick syrup, stirring with a wooden spoon all the time. This, of course, prevents it from boiling over, and enables the scum to be removed as it rises to the top. When the mixture has become a thick syrup remove it from the pan and rub it through a piece of muslin, so as entirely to strain off the pips. This done, turn the now clear syrup back again into the pan, adding a little more sugar. Boil up again for a few minutes until the sugar "cooks"; then turn into jam-jars, which must have been made thoroughly clean and dry. Set the mixture in them to cool, and tie them down with bought jam-top covers. The concoction, whose name may be revealed as strawberry jelly, will keep for any length of time if stored in a cool place. It may be served in place of jam, and proves a delicious and, for some unexplained reason, uncommon appetite-tempter for invalids who cannot eat fruit with pips, and mostly only know even of red-currant jelly as a relish for saddle of mutton. It is, however, more difficult to make than red-currant jelly, because of its fine pips. Those responsible for sick-room cookery, however, are mostly deterred not by the presence of difficulties, but by an absence of new ideas.

### A FARTHING FUND.

At the annual Flower Service recently held at All Saints' Church, Highams Park, at which incidentally the collection was also given to the local hospital, and this year beat previous records—a noteworthy gift was an envelope marked "For the Walthamstow Hospital, with good wishes," and which proved to contain the sum of six shillings, the result of a farthing collection. This was made by the members of the Girls' Guild, and it is interesting to learn that a "Hospital Farthing Fund" has always been a prominent feature of that organisation. In the modern world the widow's mite is the basic power of the millionaire's millions. All huge businesses, from groceries and pin-makers to the halfpenny daily and nightly Press, rely on it. But hospitals have not the same numbers to which to appeal.

### ALEXANDRA DAY AT CARDIFF.

The celebration of Alexandra Day at Cardiff was necessarily postponed until July, owing to the inability of headquarters to supply a sufficient quantity of roses until after the celebration in London. The delay has not proved prejudicial, as the result is likely to prove at least as successful as either of the two preceding years. On Tuesday, 6th inst., a garden party in connection with the *fête* was given by Colonel and Mrs. Henry Lewis at their historic home, "Greenmeadow," Tongwynlais. It is probable £150 will be realised by this, but the ticket returns had not all been completed. Thursday, July 8, the day for the sale of flowers, proved perfect as regards weather conditions, and from early morning the streets and outlying districts of Cardiff were invaded by an army of 1,500 daintily-clad flower-sellers, who sold during the course of the day some 150,000 roses. The Lady Mayoress, Mrs. J. T. Richards, with a selected detachment, early in the day succeeded in securing the "Cardiff Exchange," working in collusion with the President of the Chamber of Commerce, Mr. T. E. Watson. The Exchange quickly capitulated, enthusiastically handing over a record indemnity amounting to £300, Commander Edward Nicholls himself putting up £105, whilst another well-known shipowner contributed fifty guineas. The counting of the boxes later revealed that the day appealed especially to the working people, for the weight of copper coins in the boxes exceeded three tons. It is computed that King Edward VII.'s Hospital, Cardiff, will receive about £1,000 as a result of the celebration, and the Lady Mayoress and Mrs. Price Williams, the indefatigable Honorary Secretary, are to be congratulated on the result.

### NOT WANTED BY THE WAR OFFICE.

Nowadays we have to try to be quiet and do what we are told. Yet the experience is sometimes a very trying one. Probably none of us outside the sacred circle fully appreciates the difficulties of the official world, but we can hardly refrain from wonder at the doings of the strange folk who inhabit it. And we never manage to crush completely a suspicion—nourished by many experiences—that Government offices have no kindly feeling towards outside and private initiative. On the whole, in the present crisis, we must suffer in silence, for this is no time for indignant letters to the Press, and as for questions in Parliament, most people think there are too many of them already. It is the War Office again, and this time it is Belfast which is the victim. Patriotism is no monopoly of the northern city, but the sentiment, as is well known, burns there with a particularly vigorous flame and has withal a specially vigorous quality. Under its influence the local medical profession has organised a scheme which, were it not for the decision of our pastors and masters to the contrary, we should have pronounced excellent. The proposal was to organise a stationary general hospital for service at the Front, the staff to consist of some of the best known physicians and surgeons of the city. Each of these would undertake a six months' term of service, and as one group retired, others were organised to take the vacant places. Thus continuity of service could be guaranteed for the duration of the war. All of these arrangements, in the form of a definite offer, were submitted to the War Office. But the authorities will have none of it, and the scheme is frustrated by the official barbed wire. To service in series and by corporate organisation the War Office will not listen. For medical men individually the powers may perchance have attention, but for a group they have no voice. And a continuous service is a thing of nought, unless every volunteer gives a promise for twelve months. Hence Belfast is declined with thanks. Perhaps some day these mysteries will be explained.

### THE BACILLUS IN THE BOOK.

Public libraries have not infrequently been accused of disseminating infectious diseases by means of books circulated by their lending departments. At the present time full use is made of the information available through the Public Health Office concerning the local incidence of cases of infectious disease, and precautions can be taken accordingly with regard to books coming from infected houses. Authorities in fever hospitals are practically agreed that the risk of infection by means of such articles as books is a small one. With regard to pulmonary tuberculosis there is, in these days of the widespread advertisement of the tubercle bacillus, a not unnatural fear that books read by consumptives do harbour the germ in an active state for a long period. It is obvious that the pages of a book as they are perused by an "open" case of pulmonary tuberculosis must be well sprinkled with bacilli, and subsequent readers, especially if they are in the gross habit of turning the page with the aid of a wetted thumb, are liable to engulf some of these bacilli. It is therefore of great practical interest to refer to some experiments recently made by Dr. Emily Dove and by Dr. Henry Kenwood, the medical officer of health of Stoke Newington. In these experiments the books and papers used were subjected to much grosser contamination than that resulting from the use and coughing over by the average phthisical reader. Without going into the interesting details of the experiments carried out by Dr. Dove, which may be found in the *Lancet* for July 10, it will suffice to record the conclusion arrived at. This is, that there is no material risk involved in the reissue of books recently read by consumptives, unless the books are obviously soiled. Even then the risks are slight, and may be provided against by the withdrawal from circulation of any dirty books received from consumptive readers, until such books have been "quarantined" in a separate room for a month or disinfected by moist heat. Naturally, very dirty books would be withdrawn altogether. The disinfection of books by any form of heat or by chemical means is generally a very unsatisfactory process on account of the damage done to the binding, especially if of high-grade quality and material, so that it is of practical value to be assured that a month's quarantine appears to be efficacious in depriving the tubercle bacillus of its power to harm the patrons of public libraries.

### A CASE OF ANTHRAX AND A SHAVING BRUSH.

There are certain well-defined occupations in which the risk of infection by anthrax is recognised, and where precautions are taken to avoid it. Apart from these, the incidence of the disease is highly exceptional and the chain of causation may be very difficult to trace. This statement is illustrated by a recent experience at Hammersmith, where a solicitor's clerk has died from anthrax infection as demonstrated by a blood examination. How the disease was acquired it is impossible to say. The report reads that the unfortunate man noticed a "pimple" on his neck, presumably due to some slight injury in shaving, that the next day there was considerable swelling of the neck, and that anthrax bacilli were detected in the blood at the West London Hospital. One of the L.C.C. veterinary surgeons who gave evidence at the inquest suggested that the organism had been conveyed by a shaving-brush which might possibly have been made from the hair of an infected animal. There is a good deal of pretence nowadays of "antiseptic" shaving, but it is difficult to imagine that the essential parts of the performance are carried out with thoroughness, seeing that they must be conducted by those who have no training in the principles involved. It is of course hopeless to expect to avoid all the risks which existence involves, but the mere suggestion of anthrax as a possible result of shaving is enough to make us all become, if not "full of strange oaths," at least "bearded like the pard." Or, if the modern fashion must prevail, there are effective shaving-pastes which render the brush unnecessary. They are much more cleanly and sanitary than the usual method, and it is surprising they are not more widely adopted.

### ST. ANDREW'S AMBULANCE CORPS.

A second unit of 100 men for ambulance service abroad has recently been raised by the St. Andrew's Ambulance Corps. The men left Glasgow last month, and after a brief training at Aldershot will be drafted to one or other of the Expeditionary Forces. Previous to their departure they were entertained to supper by the Council of the Association, Colonel D. J. Mackintosh, M.V.O., being in the chair. A first unit of 100 men was supplied in October last at the request of the War Office, and a further request being received at the end of May, the necessary volunteers were obtained in the course of a week. At the present time more than 1,000 of the St. Andrew's Ambulance Corps are serving with the Forces at home and abroad. Since the beginning of the war nearly 25,000 sick and wounded have been carried in Glasgow on the ambulance cars of the corps, 10,000 in Edinburgh, and large numbers in Aberdeen and Dundee. The fund for sending motor ambulances to the Front has reached a total of over £70,000, and fifty-five cars were presented to the War Office in the early part of the present year. In many other directions valuable work has been done, as, for example, at the Scottish Red Cross Hospital at Rouen and the Scottish Women's Hospitals in France and Serbia.

### CHILDREN'S HOSPITALS AND THE WAR.

The influence of the war on the Belgrave Hospital for Children, Clapham Road, S.W., has been shown in a falling off in the funds, with a corresponding increase in the cost per bed of 11d. During the year, however, the whole of the forty cots were occupied, whilst only thirty-seven were available in 1913, and despite these disadvantages the year closed, states the report, with the finances in a fairly satisfactory condition. The "Childer Chaine," on which we commented soon after its inception, last year handed to the hospital funds the substantial sum of £85. The staff has undergone considerable alterations during the past twelve months, the chief being the retirement of Mr. Francis Jaffrey, senior surgeon, owing to ill-health. He has been elected consulting surgeon, whilst Mr. Lionel E. C. Norbury has been appointed to fill his position, with Mr. Chad Woodward as assistant surgeon. The hospital is situated in a district teeming with children, the wards are generally full, and the number of out-patients still increases. It is therefore to be hoped that in days of patriotic fervour and child-welfare schemes the children of Clapham Road district will not be forgotten.

### A BAD NIGHT.

A hospital officer had recently an unpleasant experience, which deserves a brief record among the incidents of this time. Happening to live in what may be called the danger zone, his private house suffered some damage. And as in his official capacity he was responsible for nearly double the beds ordinarily under his care, to say nothing of the arrangements and organisation on which this increased accommodation depended, he had his hands full, without domestic anxiety. To return home and find that one's house has been damaged through the attentions of a hostile visitant is almost as bad as to be present when the visitation took place. The officer in question had then to undergo the anxious process of being compensated. Such compensation as he was able to secure did not cover the expenses entailed upon him. That is a regrettable statement to make at a time when hospital men throughout the country have rendered such splendid service to the nation. But the fact provides another instance of the need for the British Hospitals Association to be up and doing at the present time. The opportunity lately missed by them is considered fully in our second leading article.

### THIS WEEK'S DRUG MARKET.

There is no falling off in the large volume of business notwithstanding the constant advances in prices. The most noteworthy rise in value is in the case of cod-liver oil, which has advanced by nearly 45 per cent. since the beginning of the month. Such a substantial rise in value was not expected, and certainly cannot be attributed to any abnormal falling off in supplies, since the fishing season which has just closed yielded a fair average amount of oil; it is rather due to a combination among the dealers in Norway, a renewal of the demand from Germany, and considerable buying on the part of the Russians. It is very difficult to advise buyers what to do in the circumstances, for although it might seem unwise to make purchases at the present extreme prices, it is not at all improbable that the highest figure has not yet been reached. There are at last signs of an impending decline in the prices of acetyl-salicylic acid and phenazone in consequence of the arrival of further supplies, but it is doubtful whether the decline will be very substantial. There is practically no change in the position of opium, the value of which is well maintained. In consequence of the further rise in the price of quicksilver mercurials are again dearer. Supplies of Epsom salts are expected from the United States, and at any rate a small reduction in the present high prices is probable. The price of Balsam Peru is not so firmly maintained. At the recent public sale of drugs in Mincing Lane the demand was not very brisk, although a fairly large supply of goods was offered. Senna sold at again dearer rates. Nux vomica was dearer. On the whole there were few noteworthy changes in value.

